# THE EFFECT OF SUPPLEMENTAL NUTRITION ASSISTANCE PROGRAM GENEROSITY ON DEPRESSIVE SYMPTOMS AMONG OLDER AMERICANS

**DOI:** 10.1093/geroni/igac059.2863

**Published:** 2022-12-20

**Authors:** Peiyi Lu, Katrina Kezios, Adina Zeki, Al Hazzouri

**Affiliations:** Columbia University, New York, New York, United States; Columbia University, New York, New York, United States; Columbia University, New York, New York, United States; Columbia University, New York, New York, United States

## Abstract

Food insecurity has been indicated to associate with more depressive symptoms. Supplemental Nutrition Assistance Program (SNAP) is an established anti-poverty government program that targets food insecurity. The goal of this study was to assess whether SNAP generosity, defined at the state level, impacts the relationship of food insecurity and depressive symptoms among older Americans. We linked state-level SNAP policy data to the individual-level data of older adults (aged 60+) from the longitudinal Health and Retirement Study (Nf12,801). Our study baseline was survey year 2000. A state is considered SNAP generous when it eliminates income/assets limit from its SNAP eligibility criteria. All HRS participants reported their food insecurity status and depressive symptoms biennially. Participants were followed until 2018. We conducted state fixed-effect models adjusted for demographic and health measures. Results indicate household food insecurity was associated with higher risk of elevated depressive symptoms (OR=1.83, 95%CI=1.61, 2.07) from a fixed effect logistic model, and this effect was stronger when living in non-generous states compared with generous states though confidence intervals were too wide to be conclusive (OR_interaction =1.09, 95% CI= 0.83, 1.42). The relative excess risk due to interaction (RERI) was 0.17 (95%CI= -0.30, 0.63). Our study finding suggested that there is no to weak evidence that state-level SNAP generosity may alleviate or offset the risk of depressive symptoms associated with food-insecurity.

